# A novel HIF-1α-integrin-linked kinase regulatory loop that facilitates hypoxia-induced HIF-1α expression and epithelial-mesenchymal transition in cancer cells

**DOI:** 10.18632/oncotarget.3186

**Published:** 2015-03-16

**Authors:** Chih-Chien Chou, Hsaio-Ching Chuang, Santosh B. Salunke, Samuel K. Kulp, Ching-Shih Chen

**Affiliations:** ^1^ Division of Medicinal Chemistry, College of Pharmacy and Comprehensive Cancer Center, The Ohio State University, Columbus, Ohio, USA; ^2^ Institute of Biological Chemistry, Academia Sinica, Taipei, Taiwan

**Keywords:** Hypoxia-inducible factor-1α, integrin-linked kinase, epithelial-mesenchymal transition, YB-1, Foxo3a

## Abstract

Here, we described a novel regulatory feedback loop in which hypoxia induces integrin-linked kinase (ILK) expression through a HIF-1α-dependent mechanism and ILK, in turn, stimulates HIF-1α expression through cell type- and cell context-dependent pathways. HIF-1α increased ILK via transcriptional activation. ILK increased HIF-1α levels by promoting mTOR-mediated translation in PC-3 and MCF-7 cells, and by blocking GSK3β-mediated degradation in LNCaP cells, consistent with the cell line-/cellular context-specific functions of ILK as a Ser473-Akt kinase. We show that ILK can account for the effects of hypoxia on Akt, mTOR, and GSK3β phosphorylation. Also, ILK can de-repress HIF-1α signaling through the YB-1-mediated inhibition of Foxo3a expression. In concert with HIF-1α, these downstream effectors promote epithelial-mesenchymal transition (EMT) through modulation of Snail and Zeb1. Thus, the ILK-HIF-1α regulatory loop could underlie the maintenance of high HIF-1α expression levels and the promotion of EMT under hypoxic conditions. Finally, we show that the small-molecule ILK inhibitor T315 can disrupt this regulatory loop *in vivo* and suppress xenograft tumor growth, thereby providing proof-of-concept that targeting ILK represents an effective strategy to block HIF-1α expression and aggressive phenotype in cancer cells.

## INTRODUCTION

Hypoxia is one of the hallmarks of solid tumors as the majority of tumor cells lie beyond the diffusion distance of oxygen (100–150 μm) from the tumor vasculature [1]. However, tumor cells can adapt to the low-oxygen environment by deploying a series of cellular responses, the so-called hypoxic responses, that confer aggressive, metastatic, and stem cell-like phenotypes to cancer cells [2, 3], which define hypoxia's role as a driving force during tumor progression and metastasis [4–6]. Mechanistically, these hypoxic tumor responses are mainly mediated through the master regulator hypoxia-inducible factor-1 (HIF-1) [4–6], a heterodimeric transcription factor consisting of oxygen-sensitive HIF-1α and constitutively expressed HIF-1β subunits. Under normoxic conditions, HIF-1α is unstable as it is hydroxylated on proline residues by specific prolyl hydroxylases (PHDs), leading to von Hippel–Lindau protein (VHL)-dependent ubiquitination and subsequent proteasomal degradation [5]. This oxygen-dependent degradation, however, is inhibited under hypoxic conditions which inactivate PHDs, allowing the cellular accumulation of HIF-1α. Moreover, despite a decrease in global protein translation during hypoxia, HIF-1α continues to be translated, of which the underlying mechanism, however, remains unclear [7].

Increased HIF-1α expression triggers the activation of a large array of genes involved in tumor growth, glycolytic switch, angiogenesis, and epithelial-mesenchymal transition (EMT) [6–9]. For example, HIF-1α induces the gene expression of various EMT regulators, including Twist, Snail, and Zeb1, thereby promoting a more aggressive tumor phenotype, which confers on cancer cells the ability to invade basement membranes and metastasize to distant sites. Another important HIF-1α target, lysyl oxidase, plays a crucial role in facilitating myeloid cell recruitment to form premetastatic niches [10]. Clinically, HIF-1α overexpression has been correlated with tumor grade, metastasis, and poor prognostic outcomes in many types of cancers [11].

In addition to the global effect of HIF-1α on gene expression reprogramming, hypoxia has also been reported to activate Akt and induce inactivating phosphorylation of GSK3β [12, 13]. From a survival perspective, this hypoxic response bestows on tumor cells a twofold advantage. First, Akt functions as a critical regulator of cell proliferation and survival that can confer resistance to chemotherapeutic agents or radiation. Second, Akt can upregulate HIF-1α expression by increasing mTOR-mediated protein translation and/or inhibiting GSK3β-facilitated, VHL-independent protein degradation [7, 14]. However, the mechanism by which hypoxia facilitates Akt and GSK3β phosphorylation is unclear, as this Akt activation was not associated with hypoxia-induced increases in epidermal growth factor receptor expression [13].

In this study, we report a regulatory feedback loop between HIF-1α and integrin-linked kinase (ILK) that might underlie the ability of cancer cells to maintain high levels of HIF-1α expression and to promote EMT under hypoxic conditions. Substantial evidence has demonstrated the critical role of ILK in mediating diverse oncogenic functions through a broad range of downstream effectors [15, 16]. The tumor-promoting effects of ILK, in part, is attributable to its ability to phosphorylate Ser473-Akt and GSK3β [17], as well as to induce the expression of the oncogenic transcription/translation factor Y-box binding protein 1 (YB-1) [18, 19]. Data from this and other laboratories indicate that ILK expression can be upregulated in response to hypoxia through HIF-1α-facilitated ILK gene expression [20], indicating a role for ILK as a downstream effector of HIF1α. Moreover, ILK has been demonstrated to play important roles in tumor angiogenesis and radioresistance by upregulating HIF-1α-dependent expression of vascular endothelial growth factor (VEGF) and survivin, respectively [21]. Here, we integrate these previous findings through evidence of a novel ILK-HIF-1α regulatory feedback loop, in which hypoxia induces ILK expression through a HIF-1α-dependent mechanism and ILK, in turn, stimulates HIF-1α expression through an mTOR- or GSK3β-dependent pathway. This feedback loop is capable of maintaining high levels of HIF-1α expression during hypoxia, and might also account for high levels of endogenous HIF-1α expression in PC-3 and other prostate cancer cell lines [21]. This HIF-1α-mediated ILK expression provides a mechanistic basis to account for the effect of hypoxia on Akt, mTOR, and GSK3β phosphorylation. Moreover, the ability of ILK to inhibit the expression of the tumor suppressor Foxo3a via YB-1-mediated transcriptional repression is noteworthy in light of the role of Foxo3a as a negative regulator of HIF-1α signaling [22]. These downstream effectors, in conjunction with HIF-1α, promote EMT by modulating the expression of various EMT regulators/makers, including Snail, Zeb1, E-cadherin, and vimentin. From a therapeutic perspective, targeting ILK to disrupt the HIF-1α-ILK regulatory loop represents a viable strategy to block hypoxia-induced HIF-1α upregulation and EMT, of which the proof-of-concept was provided by the ILK inhibitor T315 both *in vivo* and *in vitro*.

## RESULTS

### HIF-1α and ILK form a regulatory loop in facilitating hypoxia-induced HIF-1α expression and EMT in prostate and breast cancer cells

**Figure 1:**
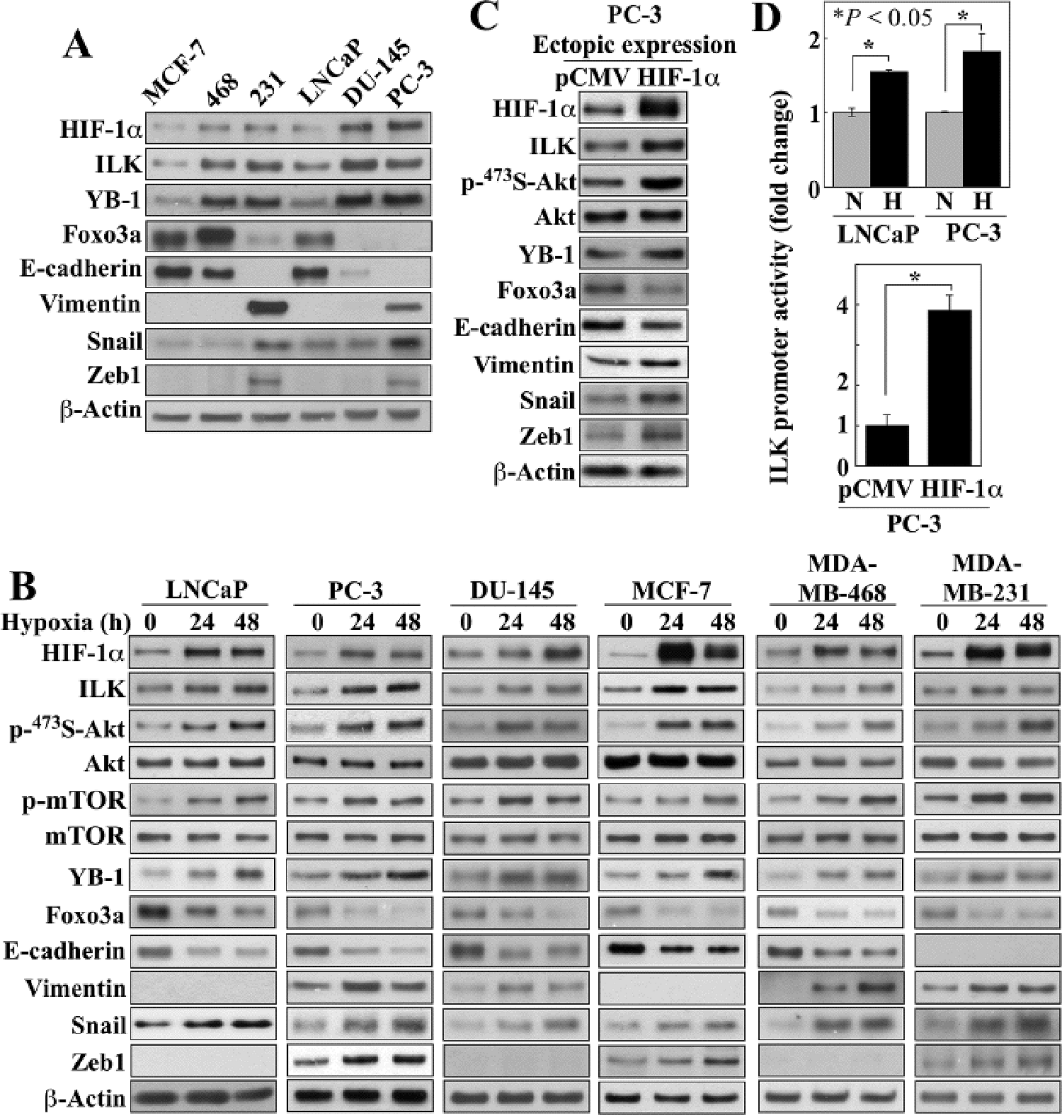
Evidence that ILK is a HIF-1α-responsive kinase in hypoxia-treated cancer cells (**A**) Western blot analysis of the expression levels of endogenous HIF-1α, ILK, YB-1, Foxo3a, and markers of EMT in breast and prostate cancer cells in 10% FBS-containing medium. (**B**) Time-dependent effects of hypoxia (0.5%) on the expression/phosphorylation levels of relevant markers in breast and prostate cancer cells. (**C**) Effect of the ectopic expression of HIF-1α on the expression/phosphorylation levels of relevant markers in PC-3 cells. (**D**) Luciferase reporter assays of the effect of hypoxia (*upper*) and ectopic expression of HIF-1α (*lower*) on ILK promoter activity in LNCaP and/or PC-3 cells. Data are presented as mean ± SD (*n* = 6). Immunoblots are representative of three independent experiments.

To shed light onto the putative role of ILK in the adaptive response to hypoxia that affords growth and survival advantages in cancer cells, we examined the effects of hypoxia on the cellular accumulation of ILK and HIF-1α, and the expression/phosphorylation levels of ILK targets (Akt, mTOR, GSK3β, and YB-1) and a series of EMT regulators/markers [the tumor suppressor Foxo3a, the epithelial marker E-cadherin, the mesenchymal marker vimentin, and the transcriptional repressors Snail and its target Zeb1 [23, 24] in breast (MCF-7, MDA-MB-468, and MDA-MB-231) and prostate (LNCaP, DU-145, and PC-3) cancer cell lines. Under normoxic conditions, the expression of ILK and YB-1 varied among these cells lines in concert with that of HIF-1α, consistent with the reported role of ILK as a hypoxia-inducible kinase [20] (Figure 1A). In addition, the cell lines with a mesenchymal phenotype (MDA-MB-231, DU-145, and PC-3), showed higher endogenous levels of HIF-1α, ILK and YB-1 than those with epithelial characteristics (MCF-7 and LNCaP). Irrespective of these differences, hypoxia concomitantly elevated the expression of these three proteins, accompanied by a parallel increase in Ser-473-Akt phosphorylation and changes in the expression of the aforementioned EMT regulators/markers in all six cell lines (Figure 1B). The role of HIF-1α in mediating hypoxia-induced upregulation of ILK signaling and EMT was demonstrated by the ability of ectopically expressed HIF-1α to mimic the effects of hypoxia in PC-3 cells (Figure 1C). Moreover, promoter luciferase reporter assays revealed that HIF-1α facilitated transcriptional activation of *ILK* gene expression in a manner similar to that of hypoxia (Figure 1D), confirming that HIF-1α regulates *ILK* gene expression in hypoxia-exposed cancer cells.

**Figure 2:**
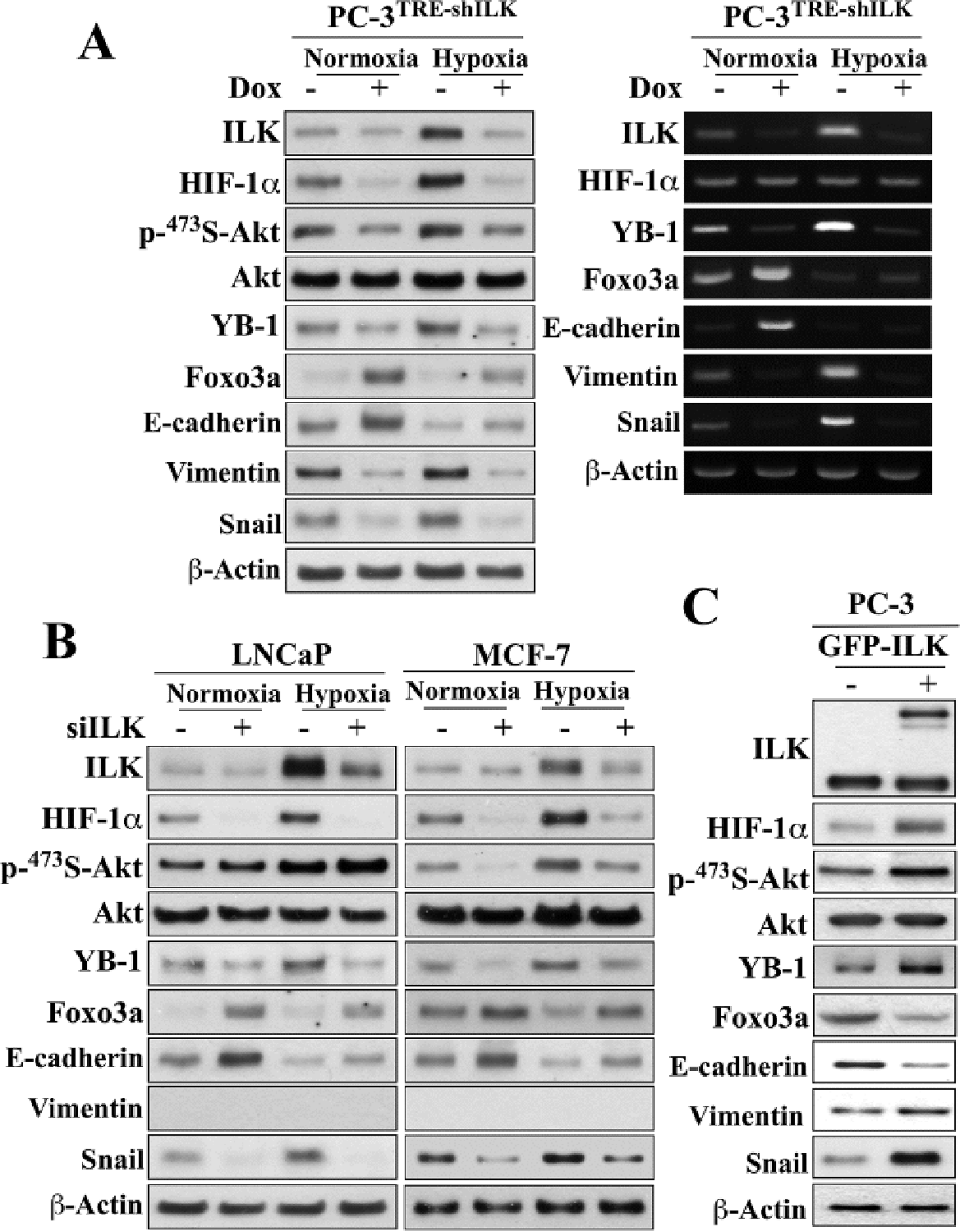
Evidence that ILK and HIF-1α form a regulatory feedback loop in regulating hypoxia-induced EMT (**A**) Effects of doxycycline (Dox)-responsive shRNA-mediated ILK knockdown on the expression and/or phosphorylation levels of ILK, HIF-1α, Akt, YB-1, Foxo3a, and EMT effectors after 48-h exposure to normoxic or hypoxic condition. *Left*, Western blot; *right*, RT-PCR. (**B**) Effects of siRNA-mediated knockdown of ILK on the expression/phosphorylation levels of the same markers as in (A) in LNCaP and MCF-7 cells. (**C**) Effects of ectopic expression of GFP-ILK on the expression/phosphorylation levels of these markers in PC-3 cells. Immunoblots are representative of three independent experiments.

Pursuant to these findings, we demonstrated that ILK, in turn, could regulate HIF-1α expression, thereby forming a positive feedback loop in maintaining HIF-1α expression, and thus EMT, under hypoxic conditions. Using a stable clone of PC-3 cells that overexpress ILK shRNA under Tet-on control (PC-3^TRE-shILK^), we showed that doxycycline-induced knockdown of ILK, as verified by parallel reduction of YB-1 expression, suppressed HIF-1α protein expression under normoxic conditions, and abrogated the hypoxia-induced upregulation of HIF-1α (Figure 2A, left). This ILK knockdown-induced suppression of HIF-1α expression occurred at the posttranscriptional level as the abundance of HIF-1α mRNA remained unchanged in response to hypoxia and/or doxycycline treatment (right). Equally important, knockdown of ILK also blocked the effects of hypoxia on Ser-473-Akt phosphorylation, as well as the protein expression of various EMT regulators/markers in PC-3 cells (Figure 2A). Similar effects were also noted in LNCaP and MCF-7 cells, with the exception of an effect on Akt phosphorylation in LNCaP cells which was unaffected (Figure 2B). Mechanistically, this discrepancy is in-line with our earlier finding that Ser473-Akt phosphorylation is regulated in a cell line-specific manner by ILK and mTORC2 in PTEN-deficient PC-3 and LNCaP cells, respectively [25]. Conversely, enforced expression of constitutively active ILK in PC-3 cells increased HIF-1α expression, accompanied by parallel changes in Akt phosphorylation and expression of EMT-associated regulators/markers (Figure 2C).

### Pharmacological inhibition of ILK by T315 abrogates hypoxia-induced HIF-1α expression via different mechanisms in different cell lines

**Figure 3:**
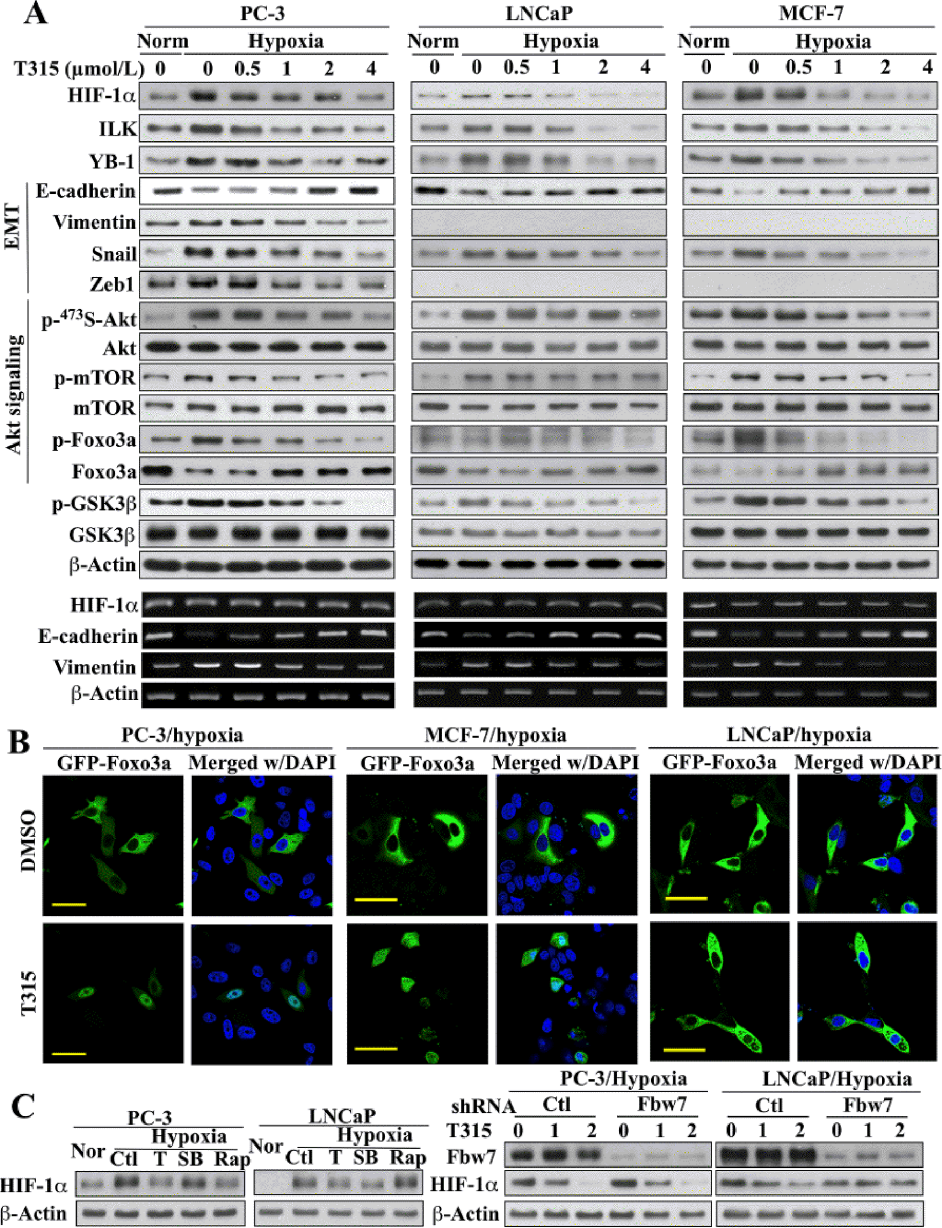
Evidence that ILK regulates HIF-1α expression through different mechanisms in PC-3 and MCF-7 cells versus LNCaP cells (**A**) *Upper*, Western blot analysis of the effects of T315 on hypoxia-induced changes in the expression/phosphorylation levels of HIF-1α, ILK, YB-1, GSK3β, and markers of EMT and Akt signaling. *Lower*, RT-PCR analysis of the corresponding effects on hypoxia-induced changes in the mRNA levels of HIF-1α and EMT markers. (**B**) Immunocytochemical analysis of the effect of T315 (1 μmol/L; 24 h) versus DMSO control on the cellular distribution of ectopically expressed GFP-Foxo3a under hypoxic conditions. Scale bar, 50 μm. (**C**) *Left*, effects of T315 (T; 2 μmol/L), SB-216763 (SB; 10 μmol/L), and rapamycin (Rap; 10 μmol/L) relative to DMSO control (Ctl) on hypoxia-induced HIF-1α expression. Nor, normoxia. *Right*, influence of shRNA-mediated depletion of FBW7 on the T315-induced suppression (1 and 2 μmol/L) of hypoxia-induced HIF-1α expression. Immunoblots and RT-PCR analyses are representative of three independent experiments.

The role of ILK in regulating hypoxia-induced HIF-1α expression and EMT was further verified by using a proof-of-concept, small molecule ILK inhibitor, T315, in PC-3, LNCaP, and MCF-7 cells. The IC_50_ values of T315 in suppressing the viability of these three representative cell lines were: PC-3, 2 μmol/L; LNCaP, 2 μmol/L; MCF-7, 2.8 μmol/L [25]. As shown in Figure 3A, inhibition of ILK kinase activity by T315 dose-dependently suppressed hypoxia-induced increases in the expression of HIF-1α, ILK and YB-1, indicative of the ability of T315 to disrupt the HIF-1α-ILK regulatory loop. T315 also reversed hypoxia-induced changes in the EMT regulators/markers, Foxo3a, E-cadherin, vimentin, and/or Snail, in all three cell lines, restoring them to levels detected under normoxic conditions (Figure 3A). Reminiscent of the results observed after siRNA-mediated ILK knockdown, T315 suppressed the hypoxia-induced phosphorylation of Ser473-Akt and its downstream targets mTOR and Foxo3a in PC-3 and MCF-7 cells, but not in LNCaP cells. Accordingly, this differential effect of T315 on the Akt-Foxo3a signaling axis was manifested by differences in the cellular distribution of Foxo3a among these cell lines (Figure 3B). In PC-3 and MCF-7 cells, the suppression of hypoxia-induced Akt activation and Foxo3a phosphorylation by T315 (Figure 3A) was accompanied by the nuclear localization of Foxo3a (Figure 3B). In contrast, Foxo3a remained sequestered in the cytoplasm in T315-treated LNCaP cells (Figure 3B), reflecting the inability of T315 to inhibit Akt/Foxo3a phosphorylation. Nonetheless, T315 was able to downregulate GSK3β phosphorylation in all three cell lines (Figure 1A) as GSK3β represents a direct target of ILK [26].

Consistent with findings in ILK-knockdown cells, HIF-1α mRNA abundance was unaffected by treatment with T315 (Figure 3A, lower), suggesting that this drug effect was mediated at the posttranscriptional level. Mechanistically, two ILK-dependent pathways might be involved in regulating HIF-1α protein expression: mTOR-mediated translation [27, 28] and GSK3β-facilitated protein degradation [29–31]. In light of ILK's role in regulating Akt/mTOR signaling in PC-3 and MCF-7 cells, but not in LNCaP cells, we reasoned that T315 suppressed hypoxia-induced HIF-1α expression in PC-3 and MCF-7 cells by inhibiting mTOR-mediated translation, while GSK3β-mediated degradation represented a major mechanism in LNCaP cells. This premise was corroborated by the differential effects of the mTOR inhibitor rapamycin and the GSK3β inhibitor SB-216763 on HIF-1α expression in hypoxia-treated PC-3 versus LNCaP cells (Figure 3C, left). Rapamycin blocked hypoxia-induced HIF-1α upregulation in PC-3, but not in LNCaP cells. Conversely, SB-216763 suppressed this hypoxia response in LNCaP, but not in PC-3 cells.

The involvement of GSK3β-facilitated proteolysis was further confirmed by the ability of siRNA-mediated knockdown of Fbw7, an E3 ligase responsible for HIF-1α degradation [29, 31], to abolish the suppressive effect of T315 on hypoxia-induced HIF-1α upregulation in LNCaP cells (Figure 3C, right). This protective effect, however, was not noted in PC-3 cells, which, together with the aforementioned SB-216763 data (left panel), argue against the involvement of GSK3β as an intermediary effector of ILK-induced HIF-1α expression in PC-3 cells.

### T315 increases Foxo3a expression by targeting YB-1-mediated transcriptional repression

The ability of T315 to reverse the suppressive effect of hypoxia on Foxo3a was noteworthy. In addition to its known function as a tumor suppressor [32], Foxo3a has been shown to inhibit HIF-1α transcriptional activity by competing for p300 binding during vascular development [22], and to suppress EMT, in part, through the regulation of E-cadherin expression [33–35]. While inhibition of Akt might underlie the ability of T315 to facilitate the nuclear localization of Foxo3 (Figure 3B), the mechanism by which T315 abrogated the suppressive effect of hypoxia on Foxo3 expression (Figure 3A) remained unclear. Here, we obtained evidence that YB-1, a known ILK target [18, 19], acts as a transcriptional repressor of Foxo3a, and that inhibition of ILK by T315 blocked hypoxia-induced YB-1 upregulation, thereby abolishing YB-1-mediated transcriptional repression of Foxo3a.

**Figure 4:**
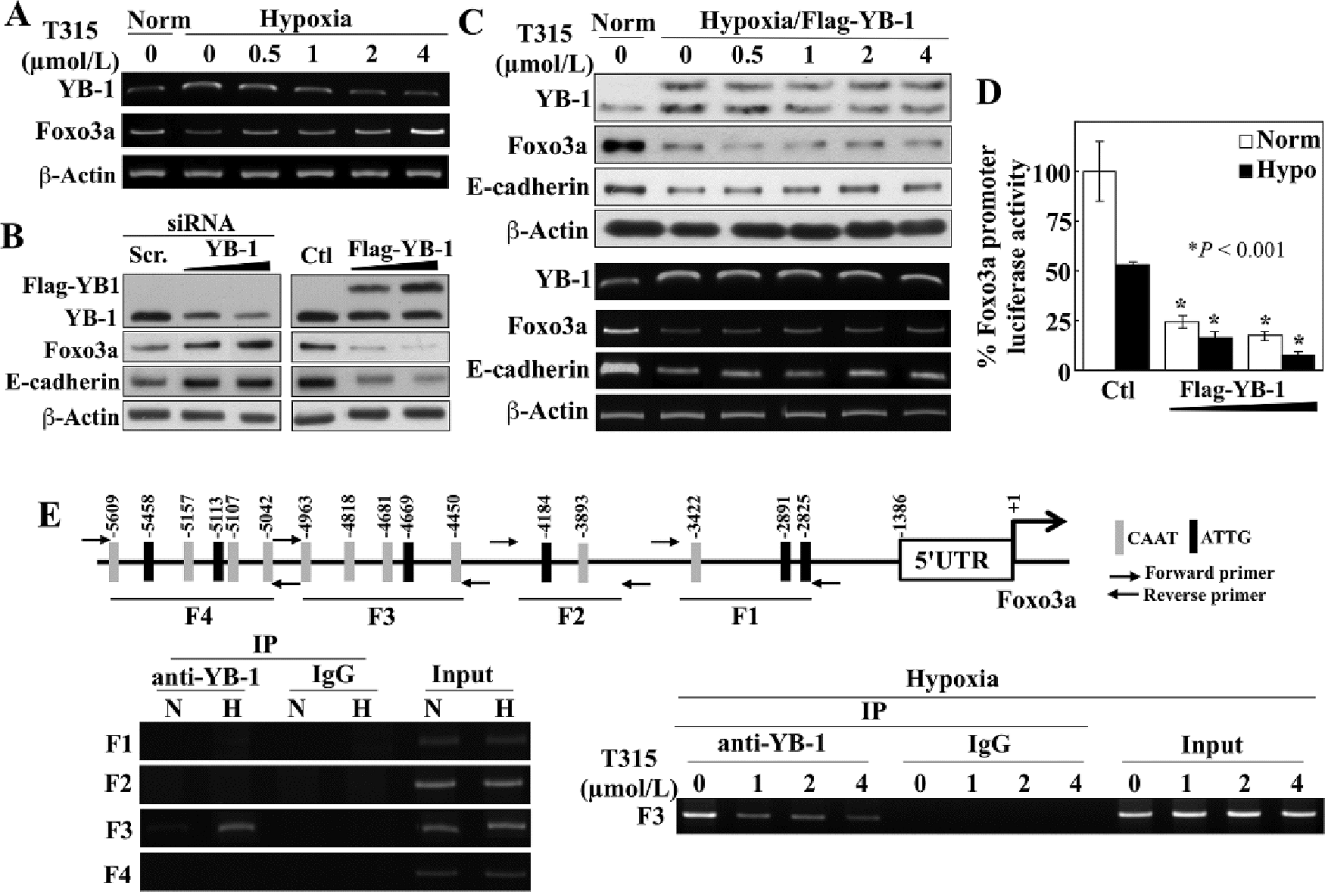
ILK inhibition by T315 reactivates Foxo3a gene expression under hypoxia by abolishing YB-1-mediated transcriptional repression in PC-3 cells (**A**) RT-PCR analyses of the effect of T315 on hypoxia-induced changes in YB-1 and Foxo3a expression. (**B**) Western blot analysis of the effect of siRNA-mediated knockdown (*left*) and ectopic expression (*right*) of YB-1 on expression of Foxo3a and E-cadherin. (**C**) Effect of ectopic expression of YB-1 on T315-induced upregulation of Foxo3a and E-cadherin expression in hypoxia-treated PC-3 cells. *Upper*, Western blot; *lower*, RT-PCR. (**D**) Luciferase reporter assays of the effect of ectopically expressed YB-1 on *Foxo3a* promoter activity under normoxic and hypoxic conditions. Data are presented as means ± S.D. (*n* = 6). *p < 0.001, compared to the respective controls. (**E**) *Upper*, depiction of 4 regions (F1–4) in the *Foxo3a* gene promoter containing putative YB-1 binding elements (indicated by vertical bars). *Lower left*, ChIP analysis of selective YB-1 binding to different regions of the *Foxo3a* promoter in response to hypoxia. N, normoxia; H, hypoxia. *Lower right*, ChIP analysis of the effects of T315 (24 h) on hypoxia-induced YB-1 binding to the F3 region of the *Foxo3a* promoter.

Consistent with our Western blot data (Figure 3A), RT-PCR analysis showed an inverse relationship between YB-1 and Foxo3a mRNA levels in response to T315 in hypoxia-exposed PC-3 cells (Figure 4A). The putative role of YB-1 as a transcriptional repressor of *Foxo3a* gene expression was supported by the abilities of siRNA-mediated knockdown and ectopic expression of YB-1 to increase and suppress, respectively, the expression of Foxo3a and its direct target E-cadherin (Figure 4B). Moreover, enforced expression of Flag-tagged YB-1 abolished the T315-induced restoration of Foxo3a and E-cadherin expression in hypoxia-treated PC-3 cells, at both protein and mRNA levels (Figure 4C). The suppressive effect of YB-1 on Foxo3a expression was attributable to transcriptional repression, as enforced YB-1 expression reduced *Foxo3a* promoter activity under both normoxic and hypoxic conditions (Figure 4D).

Analysis of the *Foxo3a* gene promoter identified multiple putative YB-1 binding sequences (a.k.a., inverted CAAT-box sequence or Y-box) in different regions of the promoter (F1–4) (Figure 4E, upper). Chromatin immunoprecipitation (ChIP) analysis demonstrated that hypoxia promoted the selective binding of YB-1 to the F3 region (lower left), and that T315 abrogated this binding (lower right). These data support our hypothesis that T315 reactivates *Foxo3a* gene expression in hypoxia-treated cancer cells by reducing the expression of YB-1 which acts as a transcriptional repressor of Foxo3a.

### T315 suppresses hypoxia-induced Snail upregulation

**Figure 5:**
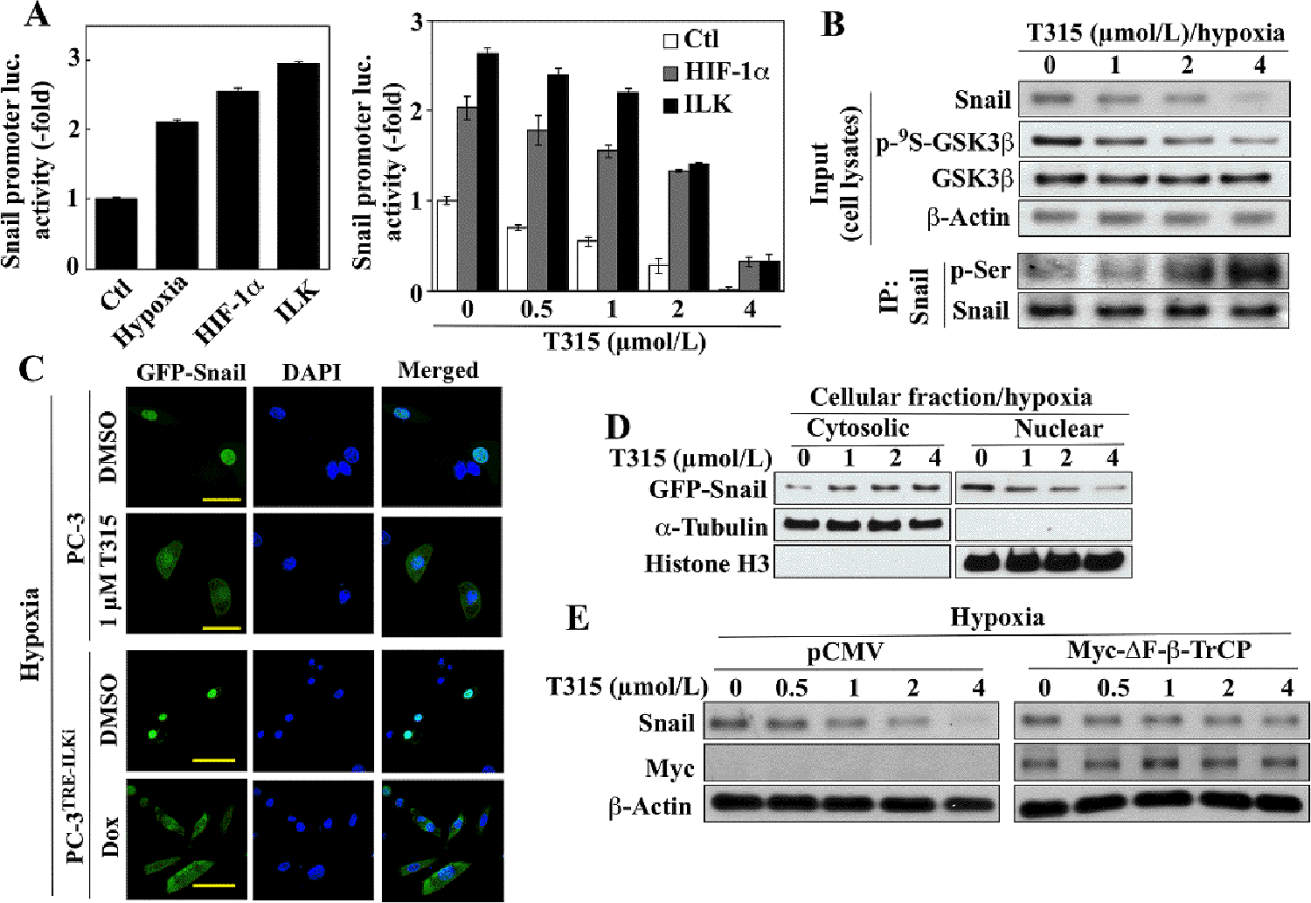
Evidence that ILK regulates Snail expression through multiple mechanisms in hypoxic conditions (**A**) Luciferase reporter assays of the effects of (*left*) hypoxia and ectopic expression of HIF-1α and ILK on *Snail* promoter activity, and (*right*), the ectopic expression of HIF-1α and ILK on T315-induced suppression of *Snail* promoter activity in PC-3 cells. Data are presented as means ± S.D. (*n* = 6). (**B**) Effect of T315 on the expression/phosphorylation levels of Snail and GSK3β in hypoxia-treated PC-3 cells. *Upper*, Western blot; *lower*, immunoprecipitation. (**C & D**) Effect of ILK inhibition on cellular distribution of ectopically expressed GFP-tagged Snail under hypoxic conditions. (C) Immunocytochemical analysis of T315-treated PC-3 and doxycycline (Dox)-treated PC-3^TRE-ILKi^ cells. Scale bar, 50 μm. (D) Western blot analysis of cytoplasmic and nuclear fractions from T315-treated PC-3 cells. Histone H3 and α-tubulin were used as internal markers for the nucleus and cytoplasm, respectively. (**E**) Western blot analysis of the protective effect of ectopic Myc-ΔF-β-TrCP expression on T315-mediated degradation of Snail in hypoxia-treated PC-3 cells. Immunoblots are representative of three independent experiments.

In addition to Foxo3a upregulation, the ability of T315 to suppress the EMT master regulator Snail and its target Zeb1 is noteworthy. The overexpression of ILK was reported to increase gene transcription of Snail in human colon carcinoma cells [36]. Here, we obtained evidence that T315 regulates the expression and cellular fate of Snail in hypoxia-treated cells through several distinct mechanisms. First, recent reports indicate that Snail expression is regulated by HIF-1α [37, 38] and YB-1 [39]. Accordingly, ectopic expression of HIF-1α or ILK in PC-3 cells mimicked the stimulatory effect of hypoxia on *Snail* promoter activity (Figure 5A, left) and partially protected cells against the suppressive effect of T315 on this activity (right). Second, GSK3β has been shown to phosphorylate Snail, leading to its cytoplasmic sequestration and degradation [40]. Consistent with this finding, T315-mediated suppression of hypoxia-induced Snail expression was associated with the dephosphorylating activation of GSK3β, and the consequent increase in Snail phosphorylation (Figure 5B). Immunofluorescence analysis indicated that inhibition of ILK, by either T315 or siRNA-mediated repression, resulted in the nuclear to cytoplasmic translocation of green fluorescent protein (GFP)-tagged Snail (Figure 5C). This finding was corroborated by Western blot analysis of the cytosolic versus nuclear distribution of GFP-Snail using anti-GFP antibodies (Figure 5D). Finally, as β-TrCP is the E3 ligase responsible for GSK3β-facilitated Snail degradation [40], enforced expression of ΔF-β-TrCP, an F-box-deleted, dominant-negative mutant form of β-TrCP [41], protected hypoxia-exposed PC-3 cells against T315-induced Snail downregulation (Figure 5D).

### T315 inhibits hypoxia-induced invasive phenotype

**Figure 6:**
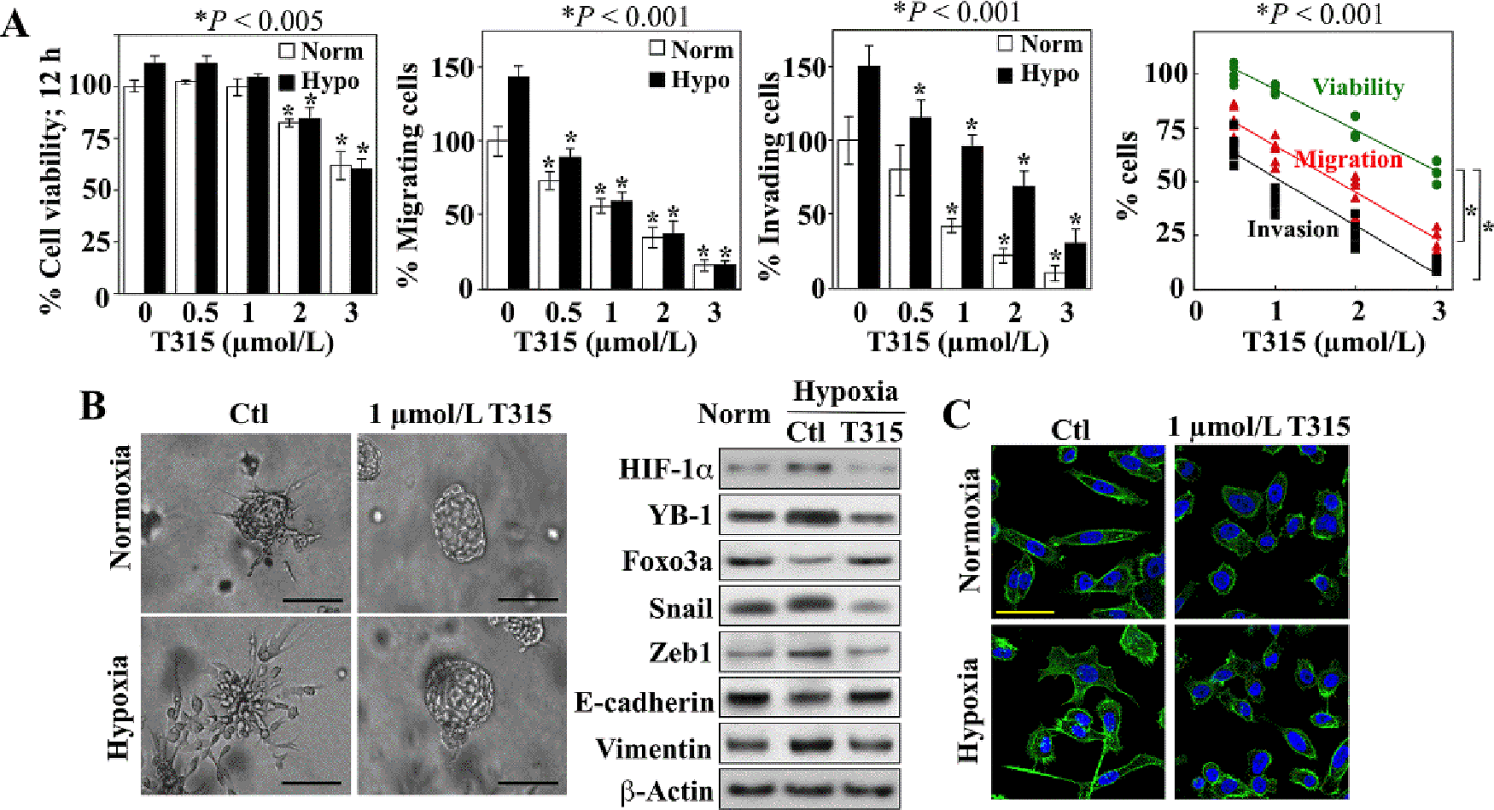
Suppressive effects of ILK inhibition on hypoxia-induced aggressive phenotype in PC-3 cells (**A**) Effects of T315 on cell viability (*left*), migratory activity (*left center*), and invasiveness (*right center*) after 12 h of treatment. Data are presented as mean ± S.D. (*n* = 6). *Right*, trend analysis of the rates of decrease in the viability, migration, and invasion of the T315-treated PC-3 cells. Individual data points from each treatment group in each assay are shown. (**B**) *Left*, photomicrographs of PC-3 colonies after growth in BME under normoxic or hypoxic conditions in the presence of T315 or DMSO control (Ctl). Scale bar, 100 μm. *Right*, Western blot analysis of the expression of HIF-1α, YB-1, Foxo3a, and EMT effectors in PC-3 cells grown in monolayers under normoxic conditions (Norm) versus that of the invasive colonies described in the left panel. (**C**) Effects of T315 versus DMSO control (Ctl) on the F-actin cytoskeleton in PC-3 cells after 24 h of treatment. Scale bar, 50 μm.

T315 inhibited PC-3 cell migration and invasion *in vitro* in a dose-dependent manner under both normoxic and hypoxic conditions (Figure 6A, center two panels). Although treatment with T315 under the same experimental conditions also caused a dose-dependent reduction in cell viability (left panel), the inhibition of cell motility and invasion was not attributable to cell death as the concentration-dependent rates of decrease in invasion and migration were significantly greater than that in viability (right panel). In addition, the suppressive effect of T315 on the metastatic potential of PC-3 cells was interrogated using the three-dimensional (3-D) colony formation assay, which is frequently used to assess metastatic capacity [42]. PC-3 cell colonies that formed under normoxic conditions were characterized by round spheroid morphology with few protrusions (Figure 6B, left). Colonies generated under hypoxic conditions exhibited stellate projections that bridged multiple colonies, indicating an invasive phenotype, which was blocked by T315, as indicated by the loss of stellate morphology. Western blot analysis of cell lysates collected from these colonies confirmed the ability of T315 to suppress hypoxia-induced changes in EMT-associated markers, restoring their expression to levels comparable to, or even lower than, those observed under normoxic conditions (Figure 6B, right). In addition, staining with FITC-conjugated phalloidin revealed the induction of pseudopod formation in hypoxia-exposed cells, which was blocked by T315 (Figure 6C). Together, these findings suggest that ILK inhibition by T315 suppressed the metastatic phenotype of PC-3 cells.

### T315 suppresses the HIF-1α-ILK regulatory loop and growth of PC-3 xenograft tumors *in vivo*

**Figure 7:**
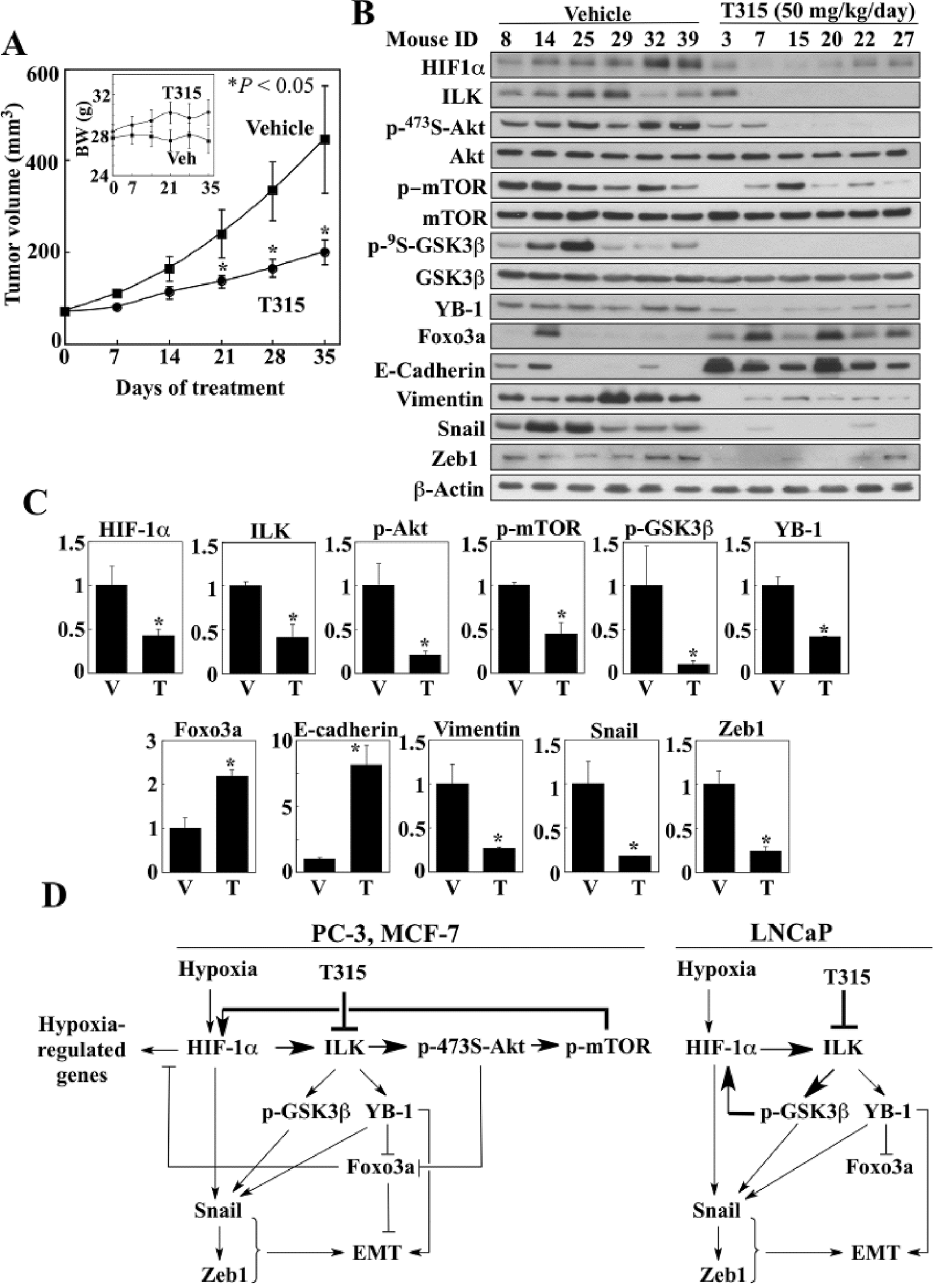
Effect of ILK inhibition by treatment with T315 on PC-3 xenograft tumors (**A**) Effect of T315 (p.o., 50 mg/kg, once daily) on PC-3 tumor growth in nude mice (*n* = 8). *Inset*, average body weights of each treatment group during the study. Data are presented as means ± S.E. (*p < 0.05 compared to vehicle). (**B & C**) Western blot analysis of the phosphorylation/expression levels of relevant markers in six representative tumors from each treatment group. (B) Immunoblots. (C) Relative expression levels of the various markers based on densitometric quantitation of band intensities. Data are presented as means ± SD (*p < 0.05 compared to vehicle). T, T315; V, vehicle. (**D**) Diagrams depicting the mechanisms by which ILK inhibition regulates HIF-1α expression and reverses the mesenchymal phenotype of cancer cells under hypoxic conditions.

In light of the high expression levels of both HIF-1α and ILK in PC-3 cells relative to the other cell lines examined and the sensitivity of PC-3 xenograft tumors to ILK inhibition [19], the PC-3 xenograft tumor model was selected to assess the ability of T315 to disrupt the HIF-1α-ILK regulatory loop *in vivo*. Daily oral administration of T315 (50 mg/kg) to tumor-bearing mice was well tolerated as no loss of body weight (Figure 7A, inset) or other overt signs of toxicity were observed. Consistent with our previous report [19], T315 significantly suppressed PC-3 xenograft tumor growth relative to the vehicle-treated control after 35 days of treatment (means ± S.E., 199 ± 27 versus 455 ± 116 mm^3^) (Figure 7A). This tumor suppression was associated with decreased intratumoral expression of HIF-1α and ILK, accompanied by parallel decreases in the phosphorylation/expression levels of ILK's downstream targets (Akt, mTOR, GSK3β), as well as increased epithelial (Foxo3a, E-cadherin) and decreased mesenchymal (YB-1, vimentin, Snail, Zeb1) markers (Figure 7B and 7C). Together, these findings suggest that ILK inhibition by a small-molecule agent reversed the mesenchymal phenotype of PC-3 tumors by disrupting the HIF-1α-ILK feedback loop.

## DISCUSSION

In light of the critical role of HIF-1α in regulating hypoxia-induced adaptive cellular responses that promote aggressive phenotype, targeting HIF-1α expression/transcriptional activity represents a therapeutically relevant strategy to overcome drug resistance and metastasis [43]. Evidence indicates that HIF-1α expression is tightly regulated during hypoxia through a complex network of pathways governing its transcription, translation, and protein stability [7]. In this study, we identified a regulatory feedback loop between HIF-1α and ILK that contributes to the maintenance of high levels of HIF-1α expression via a VHL-independent mechanism, and thereby supports the development of an aggressive phenotype and the survival of cancer cells in the oxygen-deprived tumor microenvironment. Specifically, in the initial phase of hypoxia, reduced oxygen levels result in greater abundance of HIF-1α through inhibition of PHDs and consequent protein stabilization. This cellular accumulation of HIF-1α increases the gene expression of ILK via transcriptional activation. ILK, in turn, upregulates the expression of HIF-1α through two distinct pathways in a cell line- or cellular context-specific manner (Figure 7D). Using the ILK inhibitor T315 as a proof-of-concept compound, we obtained evidence that, in PC-3 and MCF-7 cells, ILK increases HIF-1α expression by promoting mTOR-mediated translation, consistent with its role in regulating HIF-1α-induced angiogenesis [7]. In contrast, LNCaP cells compensate for the inability of ILK to mediate hypoxia-induced Akt activation (Figures 2B and 3A) by blocking GSK3β-mediated proteasomal degradation, which serves as an alternative mechanism to facilitate hypoxia-induced HIF-1α upregulation. This regulatory feedback loop underlies the ability of cancer cells to maintain high HIF-1α expression in hypoxic conditions, and also explains the high endogenous levels of HIF-1α observed in PC-3 and DU-145 cells, both of which exhibit upregulated ILK expression (Figure 1A). Moreover, this HIF-1α-ILK regulatory loop functions in the context of other feedback loops that modulate HIF-1α expression. For instance, activated growth factor pathways, such as those mediated by VEGF and insulin-like growth factor, can activate mTOR, through Akt, resulting in the translation of HIF-1α, which in turn can increase growth factor synthesis [44]. Thus, the HIF-1α-ILK regulatory loop, along with this mTOR-HIF-1α positive feedback loop, can combine to drive mTOR-mediated HIF-1α expression in cells, such as PC-3 and MCF-7, in which ILK mediates Akt activation.

By acting as a core component of the regulatory loop, ILK promotes the aggressive and metastatic phenotype of cancer cells in a hypoxic setting through multiple oncogenic signaling pathways, including those mediated by Akt, GSK3β, and YB-1 (Figure 7D). We obtained evidence that these downstream effectors, in concert with HIF-1α, contribute to the multifaceted mechanisms by which ILK signaling facilitates EMT. For example, in contrast to the previous report that ILK increased transcription of the *Snail* gene in human colon carcinoma cells [36], the present study indicates that ILK promoted the upregulation of Snail via multiple pathways involving HIF-1α, YB-1, and GSK3β at different cellular levels. Specifically, while HIF-1α [37, 38] and YB-1 [39] have been shown to enhance the expression of Snail via transcriptional and translational activation, respectively, GSK3β-induced cytoplasmic translocation and proteasomal degradation underlies the suppressive effect of T315 on Snail expression (Figure 5).

Moreover, the ability of ILK to inhibit the expression of the tumor suppressor Foxo3a via YB-1-mediated transcriptional repression is noteworthy (Figure 4). First, YB-1 has been reported to play a “master-regulatory” role in many oncogenic signaling pathways through transcriptional or translational regulation of expression [45]. In light of the tumor suppressor role of Foxo3a [32], this transcriptional repression of Foxo3a is consistent with the reported function of YB-1 in protecting tumor cells from apoptosis by acting as a transcriptional repressor of the tumor suppressor gene *p53* [46] and the cell death-associated gene *Fas* [47]. Second, Foxo3a has been shown to negatively regulate HIF-1α signaling by competing for p300 binding [22]. Thus, the ability of ILK to downregulate Foxo3a expression, in conjunction with HIF-1α overexpression, plays a critical role in facilitating hypoxia-mediated tumor progression and metastasis. Third, recent evidence has attributed the posttranscriptional regulation of Foxo3a expression to the three oncokinases Akt, IKK, and ERK [48]. These kinases phosphorylate Foxo3a at different sites, leading to Foxo3a nuclear exclusion and subsequent proteasomal degradation. Thus, this YB-1-mediated transcriptional regulation offers new insights into how cancer cells suppress Foxo3a signaling.

From a clinical perspective, our findings may have implications for prostate cancer therapy. Androgen deprivation, a mainstay of treatment for advanced prostate cancer, has been shown to induce hypoxia in prostate tumors in preclinical models [49], the significance of which is underscored by clinical data showing the correlation of tumor hypoxia with biochemical failure and clinical outcome in prostate cancer patients [50, 51]. Thus, androgen deprivation therapy, which controls androgen-dependent tumor growth initially, may ultimately contribute to progression to castration resistance through the ability of hypoxia-induced HIF-1α to promote angiogenesis, EMT, metastasis and chemoresistance, as well as androgen receptor (AR) signaling [52, 53]. Therefore, targeting HIF-1α expression through ILK inhibition, in combination with androgen deprivation therapy, may be a rational therapeutic strategy to prevent or delay progression to castration-resistant prostate cancer.

Androgen deprivation has been reported to trigger EMT in prostate cancer cells [54]. While this effect was concluded to result from the alleviation of AR-mediated repression of Zeb1 expression, androgen depletion-induced EMT could also be driven by HIF-1α, as androgen deprivation caused hypoxia in prostate tumors [49]. In contrast, others have reported that activated AR promoted EMT through decreased E-cadherin expression [55] and that prostate cancer cells underwent EMT in response to androgens [56]. Interestingly, this ability of androgens to induce EMT was inversely correlated with the expression levels of AR [56]. Specifically, evaluations of endogenous, overexpressed or silenced levels of AR expression in prostate cancer cells revealed that low AR content was required for androgen-induced EMT. We reasoned that HIF-1α might underlie this differential response of low-AR and high-AR prostate cancer cells due to its interaction with AR to form a ternary complex with β-catenin that is required for hypoxia-activated AR transactivation [57]. We suggest that in cells with low AR content, such as PC-3 cells, HIF-1α induces EMT through effectors, such as Snail and Zeb1, but has limited interaction with the low abundance AR. In contrast, in cells with high AR content, such as LNCaP cells, we suggest that HIF-1α forms the aforementioned complex with β-catenin and AR to mediate hypoxia-induced AR transactivation to promote cell growth under low androgen conditions, but, as a consequence, is unavailable to readily facilitate EMT.

In summary, we obtained evidence that the HIF-1α-ILK regulatory loop plays a critical role in maintaining HIF-1α expression and regulating EMT in cancer cells via multiple mechanisms in a cell line- and cellular context-specific manner. Moreover, the effectiveness of oral T315 to disrupt this regulatory loop and reverse mesenchymal phenotype *in vivo* provides a proof-of-concept that inhibition of ILK by small-molecule agents represents a therapeutically relevant strategy to reduce the aggressive phenotype of cancer cells. Lead optimization of T315 to generate more potent derivatives for preclinical development is currently underway.

## MATERIALS AND METHODS

Descriptions of cell culture, antibodies, and agents used are provided in Supplementary Information.

### Plasmid construction and transient transfection

The genomic sequence of Snail (chr20: 48599513–48605423) was obtained from the National Center for Biotechnology Information (NCBI). The Snail cDNA was PCR-amplified from PC-3 cDNA. The amplified fragments were cloned into *EcoRI/KpnI* sites of the pEGFP-C1 vector (Clontech, Mountain View, CA) to generate pEGFP-Snail. Whole genomic DNA from PC-3 cells was used as source for the PCR-amplification of the Snail promoter fragment containing putative hypoxia response element (HRE) consensus sequences (–212 to +192) and the Foxo3a promoter fragment (–5609 to –1386), which were cloned into *KpnI/BglII* sites of the pGL3-Basic vector (Promega, Madison, MI) to generate the pGL3-Snail-p-Luc and pGL3-Foxo3a-p-Luc plasmids, respectively. Sequences of the PCR primers used are listed in Supplementary Table S1. A fragment of the human ILK promoter comprising nucleotides –334 to +180, was cloned into the pGL3-Basic luciferase reporter vector to generate pGL3-ILK-p-Luc. The plasmids encoding hemagglutinin (HA)-HIF-1α, GFP-constitutively active (CA)-ILK, Flag-YB-1, and shRNA-FBW7 were purchased from Addgene (Cambridge, MA). Lentiviral pTRIPz vectors encoding Tet/ON inducible control shRNA or ILK shRNA were purchased from Thermo Scientific (Rockford, IL).

Cells were transfected with individual plasmids by nucleofection using an Amaxa Nucleofection system (Amaxa Biosystems, Gaithersburg, MD) according to the manufacturer's instructions. Treatments were initiated at 24 or 48 h post-transfection. Expression of various plasmids was confirmed by immunoblotting analysis.

### Luciferase reporter assay

PC-3 or LNCaP cells were transfected with the pGL3-ILK-p-Luc, pGL3-Foxo3a-p-Luc or pGL3-Snail-p-Luc plasmid for 48 h, seeded into 12-well plates (1 × 10^5^ cells/well), and incubated in a hypoxia chamber maintained at 0.5% O_2_. After 24 h, cells were treated with T315 at the indicated concentrations for another 24 h under the same hypoxic conditions. The p*Renilla* Luciferase plasmid was co-transfected as the internal control for normalization. The transcriptional activity for each promoter was analyzed by adding D-luciferin to cell lysates, and the bioluminescence signal was detected with a microplate luminometer (Promega, Madison, MI).

### RT-PCR analysis

Total RNA was isolated from cells using TRIzol (Invitrogen), and reverse-transcribed to cDNA using the Omniscript RT kit (Qiagen, Valencia, CA). The PCR products were resolved by electrophoresis on 1.5% agarose gels and visualized by ethidium bromide staining. The sequences of the PCR primers used are listed in Supplementary Table S1. Cycle numbers for these target genes were as follows: HIF-1α, 28; ILK, 25; YB-1, 30; Foxo3a, 31; E-cadherin, 28; vimentin, 26; Snail, 32; β-actin, 26.

### Immunoblotting

Growing cells were harvested by scraping and lysed in SDS lysis buffer/protease inhibitor cocktail. An equal amount of protein from each sample was separated by SDS-PAGE, transferred onto a PVDF membrane, and then probed with specific antibodies, followed by incubation with HRP-conjugated secondary antibodies. Western Lighting Chemiluminescence Reagent Plus (Perkin–Elmer) was used to develop images.

### Chromatin immunoprecipitation (ChIP) assay

After crosslinking with 1% formaldehyde for 10 min at room temperature, cells were washed with ice-cold PBS three times and whole-cell extracts were prepared with ChIP lysis buffer (50 mmol/L N-2-hydroxyethylpiperazine-N-2-ethanesulfonic acid–KOH, pH 7.5, 140 mmol/L NaCl, 1% Triton X-100, 0.1% sodium deoxycholate, 2 mmol/L AEBSF, 1 mmol/L EDTA, 130 μmol/L bestatin, 14 μmol/L E-64, 1 μmol/L leupeptin and 0.3 μmol/L aprotinin). Cellular DNA fragments of approximately 500 bp in size were generated by sonication. For immunoprecipitation, aliquots of PC-3 cell lysates (1 mg protein/aliquot) were incubated with 2 μg of anti-YB-1 antibody at 4°C for 24 h on a rotary mixer. After incubation with 30 μL of protein A/G agarose beads at 4°C for an additional 2 h, immunoprecipitates were washed twice with 1 ml of ChIP lysis buffer, twice with 1 ml of a high salt ChIP lysis buffer (containing 500 mmol/L NaC1), twice with 1 ml of ChIP wash buffer (10 mmol/L Tris, pH 8.0; 250 mmol/L LiCl; 0.5% NP-40; 0.5% sodium deoxycholate; 1 mmol/L EDTA), and then twice with 1 ml of TE buffer (10 mmol/L Tris, pH 7.5; 1 mmol/L EDTA). The immunocomplexes were eluted by addition of 75 μL of elution buffer (50 mmol/L Tris, pH 8.0; 1% SDS; 10 mmol/L EDTA) and then incubated at 65°C for 10 min. After brief centrifugation and collection of resulting supernatants, the pellets were eluted again as before. The pooled supernatants were incubated at 65°C overnight in the presence of 200 mmol/L NaCl. Aliquots containing 10 μg of protein were added to 150 μL of elution buffer as the input control. Finally, DNA was isolated from samples using a PCR purification kit. The purified DNA was analyzed by PCR using primers specific for different YB-1-binding elements in the Foxo3a promoter (F1–4). The sequences of the PCR primers used are listed in Supplementary Table S1.

### Immunofluorescence imaging of F-actin cytoskeleton

After treatment with T315 or DMSO vehicle under normoxic or hypoxic conditions, PC-3 cells were stained for F-actin with Alexa Fluor 488-conjugated phalloidin and images were obtained with an Olympus FV1000 confocal microscope (Olympus Corp., Japan) using the 40× oil immersion lens. Procedures followed those reported previously [35].

### *In vitro* migration and invasion assays

Assays were performed in typical Boyden chamber systems using Falcon™ cell culture inserts (8 μm pore size) in a 24-well format (BD Biosciences) as previously reported [35], except that PC-3 cells were seeded into the upper chambers at 2 × 10^4^ cells and 1 × 10^5^ cells per well for the migration and invasion assays, respectively, and incubations for both assays were performed for 12 h under normoxic or hypoxic conditions. All experiments were performed three times.

### Three-dimensional colony formation assay

Cells were cultured in growth factor-depleted three-dimensional Cultrex Basement Membrane Extract (BME) (Trevigen, Gaithersberg, MD) as previously reported [35], except that cells were cultured under normoxic or hypoxic conditions for 9 days.

### PC-3 xenograft tumor model

To assess the effects of T315 in tumor-bearing mice, the PC-3 xenograft tumor model was used as we previously reported [19]. Briefly, xenograft tumors were established in male athymic nude mice (5–7 weeks of age; Hsd: Athymic nude-*Foxn1^nu^*, Harlan Laboratories, Indianapolis, IN) by subcutaneous injection of 1 × 10^6^ PC-3 cells in a total volume of 0.1 ml of serum-free medium containing 50% Matrigel (BD Biosciences). Mice with established tumors (68.1 ± 16.1 mm^3^) were randomized to two groups (*n* = 8) receiving single daily treatments of T315 at 50 mg/kg or vehicle (10% DMSO/0.5% methylcellulose/0.1% Tween 80 in sterile water) for 35 days by oral gavage. Tumor volumes were calculated from weekly caliper measurements using a standard formula (volume = width^2^ × length × 0.52). Body weights were measured weekly. At terminal sacrifice, tumors were harvested, snap-frozen in liquid nitrogen, and stored at –80°C until used for analysis of biomarkers. All experimental procedures using live animals were conducted in accordance with protocols approved by The Ohio State University Institutional Animal Care and Use Committee.

### Statistical analysis

Student's *t*-tests and one-way ANOVA were used to compare differences among treatment groups. Differences were considered significant at *p* < 0.05. Statistical analyses were performed using SPSS software (SPSS Inc., Chicago, IL) and SAS 9.3 software (SAS, Inc. Cary, NC).

## SUPPLEMENTARY MATERIALS AND METHODS


