# Supplementary material for: A novel HIF-1α-integrin-linked kinase regulatory loop that facilitates hypoxia-induced HIF-1α expression and epithelial-mesenchymal transition in cancer cells
Source: Oncotarget. 2015 Mar 16;6(10):8271–85. doi: 10.18632/oncotarget.3186 (PMC4480751; doi:10.18632/oncotarget.3186)
Supplement: Supplementary file 1 [file oncotarget-06-8271-s001.pdf]

SUPPLEMENTARY MATERIALS AND METHODS

Cell culture

All cancer cell lines were purchased from the American Type Culture Collection (Manassas, VA). All cells were used in fewer than six months of continuous passage. Media used for the maintenance of these cell lines, all purchased from Life Technologies (Grand Island, NY), were as follows: DMEM/F-12 for MCF-7; DMEM for MDA-MB-468 and MDA-MB-231; RPMI 1640 for LNCaP, DU-145, and PC-3; all of which were supplemented with 10% FBS and penicillin-streptomycin. Cells were incubated at 37°C in a humidified incubator containing 5% CO_2. For hypoxia experiments, cancer cells were seeded on 6-cm culture dishes (5×10^5 cells/dish) for 24 h before transfer to a hypoxia chamber (Anaerobic System PROOX model 110, BioSpherix, Lacona, NY) maintained at 0.5% O_2, 5% CO_2 and 94.5% N_2 for different durations.

Antibodies and agents

Antibodies used in this study and their sources were as follows: Mouse monoclonal antibodies: Snail and Myc-tag (Cell Signaling Technology, Beverly, MA); E-cadherin (BD Biosciences, San Jose, CA); GFP (Santa Cruz Biotechnology, Santa Cruz, CA); β-TrCP (Invitrogen, Grand Island, NY); β-actin (MP Biomedicals, Irvine, CA). Rabbit antibodies: HIF-1α, ILK, p-Ser253-Foxo3a, vimentin, YB-1, Zeb1, p-Ser2448-mTOR, mTOR, p-Ser473-Akt, p-Thr308-Akt, Akt, p-Ser9-GSK-3β, and GSK-3β (Cell Signaling); Foxo3a (Santa Cruz); Alexa Fluor dye-conjugated phalloidin (Alexa Fluor 488; Invitrogen); anti-p-Ser antibody (Abcam, Cambridge, MA). Goat antibodies: Histone H3 (Santa Cruz); anti-rabbit IgG-horseradish peroxidase (HRP) and anti-mouse IgG-HRP (Jackson ImmunoResearch Laboratories, West Grove, PA). T315 was synthesized in-house as previously described (1).

REFERENCE

1. Lee SL, Hsu EC, Chou CC, Chuang HC, Bai LY, Kulp SK, et al. Identification and characterization of a novel integrin-linked kinase inhibitor. J Med Chem. 2011; 54:6364–6374.

SUPPLEMENTARY TABLE

Supplementary Table S1: Primer sequences

Real-time PCR		
**Target**	**Forward (5'→3')**	**Reverse (5'→3')**
HIF-1α	TGGACACTGGTGGCTCACTA	ATGCTACTGCAATGCAATGG
ILK	GACATGACTGCCCGAATTAG	CTGAGCGTCTGTTTGTGTCT
YB-1	TTGGGAACAGTAAAATGGTTCAAT	CTGCTTCTGTCTCTTTGCCATCTT
Foxo3a	TCTACGAGTGGATGGTGCGTTG	GTAGAGCATGGGCGAGAGAG
E-cadherin	CAGCGTCAACTGGACCATTG	CCACCGTTCTCCTCCGTAGA
vimentin	GACAATGCGTCTCTGGCACGTCTT	TCCTCCGCCTCCTGCAGGTTCTT
Snail	TTCCAGCAGCCCTACGACCAG	GCCTTTCCCACTGTCCTCATC
β-actin	AGCGAGCATCCCCCAAAGTT	GGGCACGAAGGCTCATCATT
**ChIP**		
**Target (Foxo3a promoter fragment)**	**Forward (5'→3')**	**Reverse (5'→3')**
F1	GCGGCGGGAAACCGTCGTCT	CCGCCCCCCAGCAAATAATA
F2	TGAATCTTTGACTCCTCCAC	GCACCTCTCCTCTCCTTGTA
F3	GAACAAGGAGGCCTACAAGA	ACTTGAGGGTATAAGACATG
F4	TAGCTGTTTGGTACACGGTG	TCTTGTAGGCCTCCTTGTTC
**Plasmid construction**		
**Target**	**Forward (5'→3')**	**Reverse (5'→3')**
Snail	CGGGATTCCATGCCGCGCTCTTTCCTCGTC	GGGGTACCTCAGCGGGGACATCCTGAGCA
Snail promoter (−212 to +192)	GGGGTACCAGTGGCCTTCGGCGGAGACGA	GAAGATCTATCCTGTGACTCGATCCTGGC
Foxo3a promoter (−5609 to −1386)	GGGGTACCTAGCTGTTTGGTACACGGTG	GAAGATCTAGCAGCACAAAGTTATAGAC
